# Effect of Motor Imagery Training on Motor Learning in Children and Adolescents: A Systematic Review and Meta-Analysis

**DOI:** 10.3390/ijerph18189467

**Published:** 2021-09-08

**Authors:** Frank Behrendt, Valerie Zumbrunnen, Lynn Brem, Zorica Suica, Szabina Gäumann, Carina Ziller, Ulrich Gerth, Corina Schuster-Amft

**Affiliations:** 1 Reha Rheinfelden, Research Department, 4310 Rheinfelden, Switzerland; valerie@zumbrunnen.be (V.Z.); lynn_brem@hotmail.com (L.B.); z.suica@reha-rhf.ch (Z.S.); s.gaeumann@reha-rhf.ch (S.G.); c.ziller@reha-rhf.ch (C.Z.); u.gerth@reha-rhf.ch (U.G.); c.schuster@reha-rhf.ch (C.S.-A.); 2 Department of Engineering and Information Technology, Bern University of Applied Sciences, 3400 Burgdorf, Switzerland; 3 Department of Health Professions, Bern University of Applied Science, 3007 Bern, Switzerland; 4 Department of Sport, Exercise and Health, University of Basel, 4052 Basel, Switzerland; 5 Department of Medicine D, Division of General Internal Medicine, Nephrology, and Rheumatology, University Hospital Münster, 48149 Münster, Germany

**Keywords:** motor imagery training, mental practice, PETTLEP, children and adolescents, randomized controlled trial, systematic review and meta-analysis

## Abstract

*Background*: There is an urgent need to systematically analyze the growing body of literature on the effect of motor imagery (MI) training in children and adolescents. *Methods*: Seven databases and clinicaltrials.gov were searched. Two reviewers independently screened references and full texts, and extracted data (studies’ methodology, MI elements, temporal parameters). Two studies were meta-analyzed providing the standard mean difference (SDM). Selected studies were evaluated with the risk of bias (RoB) and GRADE tools. *Results*: A total of 7238 references were retrieved. The sample size of the 22 included studies, published between 1995 and 2021, ranged from 18 to 136 participants, totaling 934 (nine to 18 years). Studies included healthy pupils, mentally retarded adolescents, children with motor coordination difficulties or with mild mental disabilities. The motor learning tasks focused on upper, lower and whole body movements. SMDs for the primary outcome of pooled studies varied between 0.83 to 1.87 (95% CI, I^2^, T^2^ varied 0.33–3.10; *p* = 0.001; 0–74%; 0–0.59). RoB varied between some concerns and high risk. GRADE rating was low. *Conclusions*: MI combined with physical practice (PP) might have a high potential for healthy and impaired children and adolescents. However, important reporting recommendations (PETTLEP, TIDieR, CONSORT) should be followed. The systematic review was registered with PROSPERO: CRD42021237361.

## 1. Introduction

Motor imagery (MI) is essential in everyday life for numerous human motor activities [1]. It refers to the mental simulation of action in the absence of any evident motor output [2,3] and can be defined as a dynamic cognitive process in which an individual mentally simulates an action without the external manifestation of the motor act [4,5]. According to Jeannerod [6], MI is the representation of the action involved in the planning, execution and modulation of the movements. It does not depend on residual motor function but on an internal representation of the motor act to imagine, and can thus provide a substitute for executed movement by activating the motor network [7]. It is an accepted notion that MI provides insight into an individual’s ability to generate forward models of action that subserve a target-oriented movement [2,8,9].

Neuroimaging studies have shown a similar activation during MI compared to the activation during the actual movement [10,11,12]. The crucial role of the parietal cortex in that process was revealed by an experiment which used transcranial magnetic stimulation to produce a brief suppression of the local neural activity, which in turn resulted in an impaired accuracy of MI [13]. Imagining motor acts can furthermore also activate subcortical structures, i.e., the excitability of presynaptic interneurons, without activating alpha-motoneurons [14].

MI was initially used to improve athletic performance [15,16] and has subsequently been suggested for the rehabilitation to promote motor re-learning [17]. It has become a recognized and frequently used form of training or therapy for different purposes (cognitive, strength, and motor-related tasks) and individuals [18]. It was reported that MI may substantially enhance motor rehabilitation in patients following stroke [19,20], spinal cord injury [21], orthopedic surgeries [22,23,24] and sport injuries [25,26]. The acquisition of psychomotor skills can be promoted by MI as well [27,28,29] and ideokinetic imagery was found to have a positive influence on posture and pain level in low back pain patients [30]. However, evidence was, for instance, still found insufficient to estimate the effect of MI on gait, motor function and functional mobility after stroke compared to placebo or no intervention [31]. Nonetheless, MI has been widely supported as an effective way to enhance the actual performance of motor actions [32].

MI can be stimulated mentally by using either the kinesthetic mode, which refers to the sensation of the motor act, or the visual mode by just visualizing the movement [33,34,35]. A distinction is usually also made in terms of the perspective chosen, which can be either internal or external. The Internal perspective refers to the process of imagining a movement from the first-person perspective, as if seeing a body part in motion with one’s own eyes. In contrast, with the external perspective, it is a third-person view of oneself [36,37].

The capability to image one’s own movement can influence the performance and learning of motor tasks [38,39,40,41]. An important question that has arisen in this context in the past has been how well children are able to imagine their movements. There has been a debate about the minimum age a child would be able to perform MI tasks [4,42,43,44,45]. No definitive consensus could be found on the age at which children reach the capability to imagine movements comparable to the capability of adults. Studies in six-year-olds investigating mental rotation tasks revealed that their reaction time patterns were comparable to those of adults [46]. Mental rotation training in six- to eight-year-olds led to significant improvements in the trained mental rotation tasks. Furthermore, the mental rotation training had a general effect on spatial ability. Contradictory results have been reported regarding a transfer effect in terms of a possible positive influence of spatial training on mathematics performance [47,48]. In another study, about 60% of children aged five to six years were able to use MI, compared to adults [49]. However, Butson et al. (2014) [43] found that most of the children five to six years old included in the study were only able to perform with an accuracy of below 50% of that of adults. In general, the accuracy of performance in mental rotation tasks increases with age during childhood while children aged ten and over eleven perform similar to adults [50].

The development of MI ability has been extensively studied in children without impairments [42,50]. More recently, a few studies on MI ability in children with motor deficits such as development coordination disorder (DCD) [51,52,53] or cerebral palsy (CP) have been conducted [54]. DCD is a neurodevelopmental condition that is characterized by the inability to acquire and execute well-coordinated movements at an age-appropriate level [55], reflected by slow, effortful, inaccurate and ill-coordinated movements [56,57]. The deficits in skill learning and motor coordination in such children were suggested to be the consequence of a deficient predictive motor control and perceptual-motor coupling [57]. For children with DCD, it was proposed that the associated impairments are directly related to a diminished MI ability [41,58,59,60,61,62,63]. As MI provides insight into a person’s ability to generate a forward model of an imaged action, children with DCD may have a deficit implementing a forward model into the MI process [58].

The use of MI as a tool for rehabilitation of motor function in children and adults with DCD led to the suggestion that MI might also be a useful therapeutic tool for rehabilitation of children with CP [63]. As is the case for individuals with DCD, action planning and thus MI can be severely compromised in children suffering from hemiparetic CP [64,65]. In a recent study, Errante et al. investigated the mental chronometry paradigm to study the relationship between execution and imagination of grasping actions in children with unilateral CP, and to investigate the process underlying explicit MI ability for that action [55]. The authors provided evidence that an explicit MI ability for grasping actions was preserved in these children. Consequently, their capability to retrieve motor representations should equally be preserved. Furthermore, they suggested that the application of explicit MI training could support the development of upper limb manipulation function in the cerebral palsy rehabilitation of children [54].

There is a growing body of literature on children’s ability to perform MI tasks and on the effect of MI training in various patient populations at a young age. However, to date, a systematic review of randomized-controlled trials (RCT) on these issues is still missing in the literature. Our main aim is to conduct a comprehensive systematic review and meta-analysis including the evaluation of the methodological quality of the studies. To support evidence-based clinical decision, the present review will provide an overview on RCTs on the effect of MI training (MIT) on motor (re-)learning in children of various ages without or with deficient motor function due to different diseases.

## 2. Materials and Methods

The protocol for this review was registered with PROSPERO (International Prospective Register of Systematic Review) under the registration number CRD42021237361. The review was written and reported using the Preferred Reporting Items for Systematic Reviews and Meta-Analyses (PRISMA checklist) guideline [66].

### 2.1. Search Strategy, Selection Criteria and Process

A professional librarian of the University of Zurich conducted the systematic search on the 5 March 2021 since their inception to present in the following databases:

- Cochrane Library
- Embase
- PsycInfo
- Scopus
- Web of Science
- Cinhal
- Clinicaltrials.gov

The second author (VZ) also searched SPORTDiscus on the 15 April 2021.

The search terminology regarding MI and mental practice (MP) was based on recently published Cochrane reviews in the field of rehabilitation and a peer-reviewed systematic review protocol [31,66,67]. Furthermore, the search strategy deployed followed recommendations for searching and selecting studies designed by Cochrane for identifying RCTs and controlled clinical trials (as described in the Cochrane Handbook for Systematic Reviews of Interventions) [68] of published trials if allocation of interventions was random. Studies were included regardless of publication date, if they included children or adolescents up to 18 years of age, used any kind of motor imagery training (MIT) or MP that was movement oriented, influencing any motor skill and applied one or more control interventions.

Studies were excluded if they described interventions with animals, when full texts from authors were not available or were not formally peer reviewed. To decrease the risk of missing relevant studies, reference lists in the included studies were screened. We excluded quasi-experimental or non-randomized studies using MIT or MP with technology devices, relaxation techniques or MIT with biofeedback.

**Figure 1:**
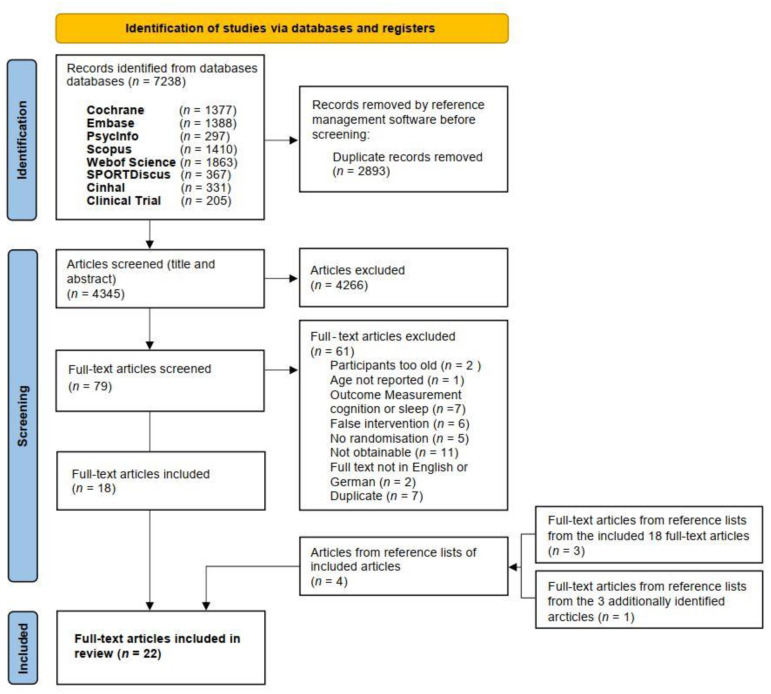
Reference selection process.

The search terms and strategy were adapted for each database. An example is provided in Table 1. Two review authors (VZ and CSA) independently screened titles and abstracts of the references obtained from the database searches, excluded irrelevant reports and removed duplicates from eligible studies. Then they retrieved the full-text articles for the remaining references. The same two review authors independently screened the full-text articles to identify studies for inclusion, and identified and recorded reasons for exclusion of the ineligible studies. In case of disagreement, a third reviewer was consulted (FB) to decide on inclusion or exclusion of the study. Finally, reference lists of the included full-text articles were screened for additional references that could yield further relevant articles. Cohen’s Kappa statistic was used to evaluate the reviewer agreement [69]. Further, a PRISMA flow diagram was generated (Figure 1).

### 2.2. Data Extraction

All authors were involved in the data extraction process. The data of each included study were independently extracted from two of the authors. When there was disagreement regarding data extraction, a third review author checked the data (CSA). A complete overview of extracted data is provided in Table 2 and Table 3. In case of incomplete data (e.g., only graphical presentations) in the selected studies, the corresponding authors were contacted to obtain the missing details. The data extraction was based on Schuster et al. (2011) [70] and focused on (1) trial-related and (2) MIT-related information:

(1) Trial-related information included: First author, year of publication, kind of randomization, blinding, sample size and study groups, age, and gender of participants, participants’ description, study setting, task to imagine, measurement events, outcomes and outcome measures, study results, number of dropouts, recognition of an included participant flow chart and the risk of bias evaluation.
(2) MIT-related information included: MI experience, MI familiarization, MI manipulation check, PETTLEP (physical, environment, timing, task, learning, emotion, perspective) approach used, MIT intervention and control interventions descriptions, MIT session and order of MIT and physical practice, location of MIT and position of the individual during MI, MI supervision and instructions’ medium, instruction individualization yes/no, perspective (internal/external), mode (kinesthetic/visual), eyes open/closed and temporal parameters: number and frequency of MIT sessions and duration and number of MI trials (per session and total).

### 2.3. Assessment of Risk of Bias and GRADE

Two reviewers (FB and CSA) assessed risk of bias within studies using the Cochrane Collaboration risk of bias (RoB) 2.0 assessment [71]. Six domains of bias were rated for every study with each domain having three rating categories. The judgement can be ‘Low’ or ‘High’ risk of bias, or can express ‘Some concerns’. Discussion between the two reviewers resolved disagreement if needed. We assessed the risk of bias according to the following six domains: (1) Randomization process, (2) Deviations from intended interventions, (3) Missing outcome data, (4) Measurement of the outcome, (5) Selection of the reported results, and (6) Overall bias.

The Grades of Recommendation, Assessment, Development and Evaluation (GRADE) was conducted by two independent rater (ZS and FB) and was used to rate the overall quality of the evidence and the strength of the recommendations for the studies and data that could be pooled in a meta-analysis [72]. In accordance with the GRADE Working Group recommendations, the evidence was classified on four levels of quality: ‘very low’, ‘low’, ‘moderate’, and ‘high quality’.

We additionally searched for study protocols to identify any deviations from the pre-planned data analysis.

**Table 2:** Characteristics of included studies.

First Author	Year	Country	Randomization and Kind of Randomization	Blinding	Study Groups	Number and Gender of Participants and per Group	Age of Participants and Per Group [Years]	Participants	Study Setting	Focus of Imagery	Measurement Events	Outcomes and Outcome Measures	Results EG vs. CG	Dropouts/ Flow Chart/ RoB Rating
Abraham [73]	2017	IL	RCT, R. stratified for age and dancing level	Investigator and participants during pre-assessment	2	Total: 25 Females EG: 13 F CG: 12	EG: 13.51 (± 0.49) CG: 13.63 (± 0.52)	Healthy dance students, at least 3 years of dancing experience	Dance studio	Elevé movement	Pretest Posttest	Ankle PF ROM in degrees Symmetry index in %: Kinematic data collected in 3-D using two digital video cameras Kinetic data were collected using the TETRAX© system MI ability: MIQ-R	No sign. between-group difference	Dropouts: 1 Flow chart: No RoB rating: SC
Asa [74]	2014	BRA	RCT, R. stratified for gender	N.R.	3	Total: 36 (F = 13, M = 23) MIT: 12 (F = 4, M = 8) PP: 12 (F = 5, M = 7) NP: 12 (F = 4, M = 8)	MIT: 9.9 ± 0.2; PP: 10 ± 0.5; NP: 9.9 ± 0.4	Healthy, right-handed pupils from a private school, average education [years]: MIT and NP: 4.4 ± 0.3 PP: 4.5 ± 0.4	N.R.	FOS: TS 4-3-2-1	BL T1: Day 1 T2: Day 4 T3: Day 7 T4: Day 28	Speed and accuracy of TS and URS: Number of correct sequences per min. recorded by a computer-monitored device attached to fingertips	MIT and PP equally effective in immediate and long-term learning, MIT leads to more efficient transfer to URS	Dropouts: N.R. Flow chart: No RoB rating: SC
Bahmani [75]	2021	IR	RCT, R. N.R.	N.R.	2	Total: 136 Males EMIT: 67 M IMIT: 71 F	Total: 10.13 ± 0.65	Healthy boys, who were naïve to the purpose of the study	N.R.	Overarm tennis ball toss with non-dominant hand	Pretest: day 1 Posttest: day 2	Tossing accuracy: Ratio of individual’s scoring for total score divided by number of completed trials MI ability: Persian MIQ-C	External imagery focus produced superior motor learning	Dropouts: No Flow chart: No RoB rating: SC
Battaglia [76]	2014	Italy	RCT, R. N.R.	Assessor	2	Total: 72 Females EG: 36 F CG: 36 F	EG: 13.8 ± 1.3 CG: 14.2 ± 1.7	Female rhythmic gymnasts, competing at national level	Summer training camp	Vertical jumps	Pretest Posttest	Vertical jump performance (FT, CT, HT, DJ, Counter Movement Jump): Optojump system MI ability—MIQ-R	FT and CT jumping parameters of the HT and DJ tests improved significantly in MIT compared to PP only.	Dropouts: N.R. Flow chart: No RoB rating: SC
Cabral-Sequeira [77]	2016	BRA	RCT R. N.R.	N.R.	4	Total: 31 (F = 15, M = 16) EG: not reported CG: not reported	Total: 13.58 ± 1.74 EG: not reported, CG: not reported	Adolescents with mild cerebral palsy	N.R.	Aiming as fast and accurately as possible at a 2 cm diameter target	Day 1: Posttest 1 Day 2: Retention test, Posttest 2, Retention 2	Movement time, Movement straightness, Frequency of sub-movement, Peak height, Average joint angular velocity, Radial error: Four opto-electronic cameras (Vicon, MX3+)	No effect associated with side of hemiparesis to achieve equivalent motor performance MIT induced faster and straighter movements in comparison with controls	Dropouts: 2 Flow chart: No RoB rating: SC
de Sousa Fortes [78]	2019	BRA	RCT, R. stratified for passing decision-making performance at BL	N.R.	2	Total: 33 Males EG: 17 CG: 16	Total: 16 ± 0.6 EG: 15.6 ± 1.9 CG: 15.6 ± 1.8	Volleyball players with at least 2 years of experience, training for 89.7 ± 4.5 min/day, 5×/week, enrolled in the U-17 Volleyball State Championship	Training site	Passing decision-making performance in volleyball	T1: 48 h before Intervention T2: 48 h after intervention	Decision-making performance—Game Performance Assessment Instrument and Decision-making index Heart rate—heart rate monitor Video recording of EG and CG sessions	Moderate positive effect of MIT on passing decision-making performance of the young volleyball players	Dropouts: N.R. Flow chart: No RoB rating: SC
de Sousa Fortes [79]	2020	BRA	RCT, R. stratified by website	Statistician	2	Total: 28 Males MIT: 14 CG: 14	MIT: 15.37 ± 0.22 CG: 15.45 ± 0.33	Tennis players (training 2 h/day, 4×/wk) enrolled in the State Tennis Championship	Tennis court	Tennis service performance	T1: 48 h before intervention T2: 48 h after intervention	Accuracy—total sum of achieved points Speed (km/h)—radar gun MI ability—MIQ- 3	MIT might be an elective strategy to enhance tennis service performance among young male tennis players	Dropouts: No Flow chart: Yes RoB rating: SC
Doussoulin [80]	2011	CL	RCT R. N.R.	Assessor	3	Total: 64 (N.R.) EG: 22 CG1: 21 CG2: 21	Total: 9–10	Elementary school students, fourth grade classes, absence of motor and/or sensory disturbances	Urban elementary school from Temuco (Chile)	Run and throw a ball towards a distant target	BL: pretest during first session T1: posttest after sixth training session	Learning—Score of Standardized Basic and Combined Movements Scale Distance reached on each ball throwing—outcome measure not mentioned	All training forms were effective in improving motor task performance, MIT and modelling were more effective to obtain a significantly higher final performance than PP	Dropouts: N.R. Flow chart: No RoB rating: High
Fekih_a [81]	2020	TUN	RCT R. N.R.	N.R.	2	Total: 38 Males MIT: 18 CG: 20	MIT: 16.9 ± 0.6 CG: 16.7 ± 0.8	Young male tennis players, volunteered, training regularly in tennis clubs for 2 h/day, on average 3×/wk	Usual training session	Tennis service	T0: 48 h before Ramadan T1: end of first week of Ramadan T2: end of second week of Ramadan T3: end of fourth week of Ramadan	Tennis service performance as a product of accuracy and speed, measured with total scores of the Service Performance Test and radar gun MI ability with MIQ-RS	MIT could be effective strategy to optimize tennis service performance during Ramadan fasting, MIT could counteract/mitigate negative and detrimental effects of fasting on tennis service performance	Dropouts: N.R. Flow chart: No RoB rating: SC
Fekih_b [82]	2020	TUN	RCT R. N.R.	N.R.	2	Total: 27 Males	EG: 16.9 ± 0.64 CG: 16.7 ± 0.59	Tennis players for at least 2 years who train in clubs for 2 h/day, 3×/wk	Tennis club sessions	Agility, speed, reaction time	T0: 48 h before Ramadan T1: end of first week of Ramadan T2: end of second week of Ramadan T3: end of fourth week of Ramadan	Agility—MAT- Agility Test Speed—ZIG-ZAG test Reaction time—video recordings MI ability—MIQ-RS for MITG only	Fasting during Ramadan reduced all performance outcomes. MIT after regular workouts may be an effective strategy to reduce the effect of fasting during Ramadan and stabilize physical performance outcomes in terms of agility, speed and reaction time for male tennis players.	Dropouts: N.R. Flow chart: No RoB rating: SC
Hemayattalab [83]	2010	IR	RCT, R. N.R.	N.R.	5	Total: 40 (N.R.) PP: 8 MIT: 8 PP + MIT: 8 MIT + PP: 8 CG: 8	Total: 12–15	Adolescents with mental retardation (AWMR)	School for mentally retarded pupils of Tehran	Basketball free throws	T1: pretest T2: posttest T3: retention test 10 days later	Basketball free throw performance: Free throw test (10 attempts) MI ability: EMG	In adolescents with mental retardation MIT by itself is less effective than PP for motor task learning enhancement, MIT + PP is more effective than MP alone	Dropouts: N.R. Flow chart: No RoB rating: SC
Kanthack [84]	2014	BRA	RCT, R. N.R:	N.R.	2	Total: 22 (N.R) EG: 11 CG:11	EG: 17.6 ± 0.5 CG: 17.6 ± 0.5	Young basketball players from one team in the junior league of the Federação Paulista de Basquete	Room near basket-ball court, and basketball court	Basket-ball free throws	T1: pretest T2: posttest	Basketball throwing performance: Scoring in 10 free throws Self-efficacy: General Perceived Self-Efficacy Scale Imagination level: customized scale ranging (0–3)	There was no significant difference between groups’ median SWC statistic indicated an 84% likelihood that MIT had a beneficial effect on performance in the first two free throws	Dropouts: N.R. Flow chart: No RoB rating: SC
Mohammadhasani [85]	2017	IR	RCT, R. N.R.	N.R.	3	Total: 36 Females MIT-CI: 11 PP-CI: 12 PP: 13	Total: 12 years old	Sixth-grade elementary school pupils	A school of Shiraz city	Kata skill (the first kata: Taikyoku Sono Ichi)	T1: pretest T2: posttest = last training session T3: 48 h after last training session T4: in a competitive condition	Kata skill learning and performance: Kata evaluation form scores 0–20	The systematic increase in the CI had highest effectiveness, MIT + PP with a systematic increase in CI had long-term positive effects on performance and learning a kata skill	Dropouts: N.R. Flow chart: No RoB rating: High
Norouzi [86]	2019	IR	RCT, R. computer-genera-ted random-number sequence	N.R.	3	Total: 45 Males MIT1: 15 MIT2: 15 CG:15	Total: 14.65 ± 1.34	Adolescent novice players in a summer camp	Football summer camp from Farhang Football School in Jahrom	Football pass skill performance	Pretest Posttest	Football pass skill performance: Mor and Christian’s test MI ability: MIQ	Among adolescent novice football players, external PETTLEP imagery led to the highest improvement in football passing skill performance	Dropouts: 0 Flow chart: No RoB rating: SC
Porretta [87]	1995	USA	RCT, R. N.R.	N.R.	2	Total: 32 (F = 17, M = 15) PP + MIT: 16 PP: 16	PP + MIT: 15 years 4 months ± 1 year 8 months PP: 14 years 7 months ± 1 years 6 months	Adolescents with mild mental retardation	N.R.	Striking with a baseball bat	During each training session on 5 consecutive days (Monday to Friday)	Timing accuracy [ms] measured as discrepancy between arrival of light stimulus at a certain location and striking the string: Bassin Anticipation Timer	Participants using PP + MIT were significantly more accurate on the striking task than participants in the PP group	Dropouts: N.R. Flow chart: No RoB rating: SC
Quinton [88]	2014	UK	RCT (matched by age), R.N.R.	N.R.	2	Total: 36 (F = 2, M = 34) EG: 18 CG: 18	Total: 9.72 ± 2.05 EG: N.R: CG: N.R.	Futsal player	Gymnasium	Dribbling and passing soccer task	Pretest Posttest	Players’ ball control and decision-making skills: Dribbling and passing soccer task MI ability: MIQ-C	MIT produced no significant improvements in imagery ability or motor task performance, Significant correlation at post-test for the MIT group between age and external visual and kinesthetic imagery ability	Dropouts: N.R. Flow chart: No RoB rating: SC
Screws [89]	1997	USA	RCT, R. N.R.	N.R.	6	Total: 30 (N.R.) EG: 10 (5 peg board; 5 pursuit rotor) PP: 10 (5 + 5) CG: 10 (5 + 5)	Total: 12.5 ± N.R.	Children with MMD	Rural School in Alabama	Cognitive-oriented task (peg board) + Skill on motoric-oriented task (pursuit rotor)	Pretest Posttest	Cognitive-oriented task: Correctly placed pegs on peg board Motoric-oriented task: Time on target for the pursuit rotor task	MIT enhanced motor performance of children with MMD on both the peg board test and the pursuit rotor task	Dropouts: N.R. Flow chart: No RoB rating: SC
Seif-Barghi [90]	2012	IR	RCT R. N.R.	Coaches	2	Total: 88 Males; U16:17; U19:18 EG: U16 = 9, U19 = 9 CG: U16 = 8, U19 = 9	Total: 18.99 ± 4.24 U16: EG 15.04 ± 0.94, CG 14.93 ± 0.63; U19: EG 17.72 ± 1.16, CG 17.31 ± 1.46	Soccer players affiliated with professional soccer clubs taking part in the national championship leagues in four age categories including U16, U19, U21 and over 21	Soccer field; educational classes in their clubs in a private room	Soccer pass performance	Pretest Posttest	Successful passing performance: Rate index after dividing by total passing counts throughout the minutes of playing presented as percentile	Players in the MIT group could observe an increase in the successful pass rate compared to CG.	Dropouts: 19 Flow chart: Yes RoB rating: SC
Simonsmeier [91]	2017	GER	RCT, R. by software before BL assessment	Judges	2	Total: 56 Females MIT first: Low Expertise: 22 High Expertise: 9 MIT last: Low Expertise: 12 High Expertise: 13	Total: 9.63 ± 2.43	Gymnasts participating in their sport between 1 and 14 years between 3.5 and 25.5 h/week	Regular training session	The cast to handstand on bars	T1: prior to first training phase T2: between two training phases T3: after second training phase	The cast to handstand on bars performance: Coding system Mental representation: SDA-M Imagery ability: SIAQ	MIT had positive effects on performance only for the high-expertise athletes in MIT last condition	Dropouts: 2 Flow chart: No RoB rating: SC
Surburg [92]	1995	USA	RCT, R. N.R.	N.R.	4	Total: 40 (F = 22, M = 18)	Total: 15.65 ± 6.65	Forty students with mild mental retardation (20 students from Indiana, 20 students from Ohio)	High school pupils	An under-hand baseball throwing task	N.R.	Successful execution of an underhand throwing task with the non-dominant arm	Two types of cognitive demands did not affect imagery MIT did improve motor performance of students with mild mental retardation	Dropouts: N.R. Flow chart: No RoB rating: SC
Takazono [93]	2018	BRA	RCT, R. N.R.	N.R.	3	Total:18 (both gender) PP: 6 PP + MIT: 6 CG: 6	Total: 9.33 ± 0.49	Right handed, healthy pupils	N.R.	Holding a plastic block and insert it in the support with the right hand	Pretest: before training Posttest: immediately after PP Retention test: 24 h after training)	Task performance: Movement time in sec reach, transport: commercial digital camera Imagery ability: MIQ-C	PP group achieved a persistent performance gain in the “transport”, but not in the “reaching” task PP + MIT group achieved persistent performance gains in both movement components No significant differences were found for CG	Dropouts: N.R. Flow chart: No RoB rating: SC
Wilson [94]	2002	AUS	RCT, R. blocked to ensure similar numbers of children within 4 percentile ranges of total impairment and similar age	Assessor	3	Total: 54 (N.R.) EG:18 PP: 18 NP: 18	Total: Range 7–12	Children with motor coordination difficulties	Six schools in Brisbane	Catching and throwing a tennis ball, Striking a softball, Jumping to a target using a two-leg take-off, balancing a ball on a bat while walking, placing objects using formboard	Pretest Posttest	Motor function: Movement Assessment Battery for Children	Imagery training, delivered in a multimedia format, can be equally effective to perceptual-motor training in developing the motor skills of children referred with coordination problems.	Dropouts: N.R. Flow chart: No RoB rating: SC

**Legend:** CG = control group; FOS = finger opposition sequence; N.R. = not reported; min = minute; MIT = motor imagery training; MITG = motor imagery training group; MITS = motor imagery training session; sec = seconds; TS = training session; WBD = weight bearing distribution; wk = week.

**Table 3:** Motor imagery training interventions of included studies.

First Author	MI Experience, MI Familiarization and MI Manipulation Check	PETTLEP Approach and MI Intervention Description	Control Interventions	MIT Session and Order	Location and Position during MIT	MIT Supervision and MI Instructions	MI Mode, MI Perspective, Eyes	Number of MITS and Intervention Duration	Trials per MITS and Total Trials
Abraham	**MI** experience: N.R. **MI familiarization:** N.R. **MI check**: Raise a hand once completing each of the imaged task Participants’ views regarding the MIT intervention Assessed with a self-administered questionnaire Focus groups 48 h after post-measurement	**PETTLEP:** yes **MITG:** Specific components of elevé performance, emphasis on ankle PF and foot movements, biomechanics, equal WBD **Progression:** Number of practiced elevé, Tempo of performance, Complexity of imagery environment	**PP:** Upper body exercises with focus on postural awareness, joint mobility and strengthening of neck, shoulders, arm, elbows, wrists. No pelvic, abdominal or lower limbs exercises **Progression**: From 6. Session use of resistance bands and light balls	**Session:** N.R., probably group **Order:** N.R.	**Location:** Dance studio **Position:** **MITG:** Lying down supine on mattress **CG:** Sitting on a chair in a circle	**Supervision:** Yes **Instructions:** Live, detailed, descriptive with changing tones of voice, and using arousing mental images	**Mode:** Visual then kinesthetic **Perspective:** N.R. **Eyes:** Closed or open, according to personal preference	**Total: 12 MITS** = 2 MITS lasting 20–25 min each per week **Duration:** 6 weeks	**Progressions:** (1) Number of mental elevé 30–80, (2) tempo of performance: 20–40 movements per min. and for static elevé 8–14 s, (3) complexity of imagery environment
Asa	**MI experience:** N.R. **MI familiarization:** N.R. **MI check:** N.R.	**PETTLEP:** N.R. **MITG:** Kinesthetic imagery of the FOS trained sequence 4-3-2-1	**PP:** Physical practice of FOS TS using the trained hand **NP:** No practice on any of the movement sequences, practiced a non-sequential painting task for the same length of time as PP and MIT	**Session:** N.R. probably individual **Order:** MIT only	**Location:** N.R. **Position:** Comfortably seated in front of a desk with supported elbows and forearms	**Supervision:** Yes **Instructions:** Live	**Mode:** Emphasized kinesthetic imagery and prevented use of visual imagery **Perspective:** N.R. **Eyes:** Closed	**Total:** 1 MITS lasting app. 25 min **Duration:** 1 day	**MITS:** Four blocks of 120 mental trials with rest intervals of 2 min between blocks **Total trials:** 120
Bahmani	**MI experience:** No experience **MI familiarization:** N.R. **MI check:** Open-ended questions	**PETTLEP:** N.R. Two different MIT groups: **MIT internal focus:** Participants were asked ‘to focus on the tossing arm’ **MIT external focus:** Participants were instructed ‘to focus on the ball’		**Session:** N.R. **Order:** PP before MIT	**Location:** N.R. **Position:** Standing, 6.1 m away from target	**Supervision:** N.R. **Instructions:** N.R.	**Mode:** N.R. **Perspective:** Internal and external **Eyes:** N.R.	**Total:** 1 MITS **Duration:** 1 day	**MITS:** Six blocks à 10 mental tosses **Total trials:** 60
Battaglia	**MI experience:** N.R. **MI familiarization:** N.R. **MI check:** 10 open-ended questions	**PETTLEP:** Yes **MITG:** (a) 3 min video observation of 3 vertical jumps (watching somebody else) + (b) 3 min: 5 mental repetitions of each of the vertical jumps (watching herself perform it) + (c) 3 min: 5 PP of each vertical jump	**CG:** Light core training (abdominal muscle exercises) and active flexibility training + 3 min: 5 PP of each vertical jump	**Session:** N.R. **Order:** MIT before PP	**Location:** N.R., probably gym **Position:** N.R.	**Supervision:** N.R. **Instructions:** N.R.	**Mode:** Visual **Perspective:** N.R. **Eyes:** N.R.	**Total:** 12 Two MITS per day for 6 days/week **Duration:** 6 weeks	**MITS:** Five mental trials of each of the 3 vertical jumps = 15 MI trials **Total trials:** 180
Cabral-Sequeira	**MI experience:** N.R. **MI familiarization:** N.R. **MI check:** Participants signaled initiation and end of trials by tapping their index finger of the resting (less affected) arm on supporting table	**PETTLEP:** N.R. **MITG:** Day 1: MIT of aiming task Day 2: PP of aiming task	**CG:** Day 1: Manipulation of a keyboard of a personal computer with less affected hand to play a game called ‘Tetris’ Day 2: PP of aiming task	**Session:** N.R. **Order:** PP before MIT	**Location:** Laboratory **Position:** Seating position on a height adjustable chair, hands relaxed and supported on table	**Supervision:** Yes **Instructions:** Live	**Mode:** N.R. **Perspective:** Internal **Eyes:** Closed	**Total:** 1 MITS lasting app. 34 min **Duration:** 2 days	**MITS:** Two sets of 5 × 10 mental trials with app. 10 min between sets **Total trials:** 100
de Sousa Fortes	**MI experience:** Yes **MI familiarization:** N.R. **MI check:** N.R.	**PETTLEP:** N.R. **MITG:** (a) Watching videos showing volleyball athletes, who were successfully executing the pass in competitive events + (b) Cognitive-general imagery executing passes during a competitive event: Construct imaginary situation in the first person Imagine the task with speed close to reality Imagine positive situations during a competition Generate emotions like in a competition	**CG:** Watched videos of advertisements related to sports equipment (e.g., caps, t-shirts, and shorts). No communication allowed during sessions	**Session:** N.R. probably group **Order:** PP before MIT	**Location:** Quiet environment in gym, close to court, Participants wore competition outfits **Position:** N.R.	**Supervision:** N.R. **Instructions:** N.R.	**Mode:** N.R. **Perspective:** Internal **Eyes:** N.R.	**Total:** 24 Three MITS/wk lasting app. 10 min **Duration:** 8 weeks	**MITS:** N.R. **Total trials:** N.R.
de Sousa Fortes	**MI experience:** Yes **MI familiarization:** N.R. **MI check:** Participants were asked to provide information about the technique adopted and the magnitude of the perceived emotions Timer to control for MI trials	**PETTLEP:** N.R. **MITG:** (a) Watching videos of tennis players that succeed at service (b) Cognitive-specific imagery: Imagine a situation in the first person Imagine the task at speeds close to reality, adopting approximately ten second intervals between each imagination of the service Imagine positive situations during a competition Replicate emotions similar to those experienced during competitions	**CG:** Watching videos about the history of the Olympics	**Session:** N.R. probably individual **Order:** PP before MIT	**Location:** Quiet environment close to the tennis court, participants wore competition outfits **Position:** N.R.	**Supervision:** Yes **Instructions:** N.R:	**Mode:** N.R. **Perspective:** Internal **Eyes:** N.R:	**Total:** 24 Three MITS/wk lasting app. 10 min **Duration:** 8 weeks	**MITS:** N.R. **Total trials:** N.R.
Doussoulin	**MI experience:** N.R. **MI familiarization:** N.R. **MI check:** N.R.	**PETTLEP:** N.R. **MITG:** MIT of the ball throwing while running task	**CG1:** Modeling condition: Watching a video recording of ball throwing performance while running being performed by an expert **CG2:** PP of ball throwing performance while running	**Session:** N.R. **Order:** PP before MIT	**Location:** N.R. **Position:** N.R.	**Supervision:** N.R. **Instructions:** N.R.	**Mode:** N.R. **Perspective:** N.R. **Eyes:** N.R.	**Total:** 6 MITS **Duration:** N.R.	**MITS:** Sixty mental trials **Total trials:** 60
Fekih_a	**MI experience:** Yes **MI familiarization:** N.R. **MI check:** Chronometer for each athlete to control the duration of mental trials of the 10 tennis service	**PETTLEP:** N.R., probably partially **MITG:** (a) PP (b) Watching videos of tennis players performing technical gestures (c) Cognitive imagery: athletes were asked to imagine themselves while performing tennis service Imagine a first-person situation Imagine the task performed at speeds close to reality, with actions interspersed by an interval of about ten seconds Imagine positive situations during a competition Reproduce emotions similar to those felt during competitions	**CG:** PP + watching videos about the history of the Olympic Games	**Session:** Individual **Order:** PP before MIT	**Location:** Quiet environment near the tennis court, participants wore competition outfits **Position:** N.R.	**Supervision:** Yes **Instructions:** N.R.	**Mode:** N.R. **Perspective:** Not clear: ‘first person situation’ **Eyes:** N.R.	**Total:** 12 Three MITS/wk lasting 10 min = app. 120 min **Duration:** 4 weeks	**MITS:** 10 mental trials **Total trials:** 120
Fekih_b	**MI experience:** N.R. **MI familiarization:** N.R. **MI check:** Chronometer for each athlete to control the duration of mental trials of the 10 tennis service	**PETTLEP:** N.R. **MITG:** (a) PP (b) Watching videos of tennis players performing technical gestures (c) MIT mode visual: Think of a situation in first person Imagine moving quickly to the next striking point Imagine changing direction in different axes Imagine fixing solid supports to the ground and quickly leaving these supports to start in motion Imagine performing powerful and precise services (d) MIT mode kinesthetic: Participants experimented and felt sensations that were evoked in a real situation of PP; participants, could speak softly or mimic the movement and used technique of body simulation of movement (e) Informal discussions with experimenter about usefulness and effects of imagery	**CG:** PP + watching videos about the history of the Olympic Games	**Session:** N.R. **Order:** PP before MIT	**Location:** Quiet environment near the tennis court, Participants wore competition outfits **Position:** N.R.	**Supervision:** Yes **Instructions:** N.R.	**Mode:** Visual before kinesthetic **Perspective:** External before internal **Eyes:** N.R.	**Total:** 12 Three MITS/wk lasting 15 min= app. 180 min **Duration:** 4 weeks	**MITS:** Ten mental trials **Total trials:** 120
Hemayattalab	**MI experience:** N.R. **MI familiarization:** Yes, 1 MI training session including internal kinesthetic imagery **MI check:** After pretest and before intervention a MIT training session including internal kinesthetic imagery and N.R.	**PETTLEP:** N.R. Three different MIT groups **MITG:** (a) Participants felt performing the task from within their own body as if they were looking out from their own eyes (b) Participants imagined the task from their own vantage point (c) Participants were told to see the rim, the backboard, the ball in their hands, and the umpire, but not the things out of their normal range of vision. Participants felt the movement, their finger gripping the ball, the stretch of their arm during the throw, the shift of weight from heels to toes, and the extension of their knees, hips and arms **MIT only:** 30× basketball free throw for 24 MITS **PP followed by MIT:** 12 TS PP of basketball free throw + 12 TS MIT basketball free throw **MIT followed by PP:** 12 TS MIT of basketball free throw + 12 TS PP basketball free throw	Two different control groups: **PP:** 30× physical practice of basketball free throw for 24 MITS **CG:** no training at all	**Session:** N.R. **Order:** Only MIT or PP in 1 TS	**Location:** N.R. **Position:** N.R.	**Supervision:** N.R. **Instructions:** N.R.	**Mode:** Kinesthetic **Perspective:** Internal **Eyes:** N.R.	**Total:** 24 30 min per MITS **Duration:** N.R.	**MITS:** 30 **Total trials:** **MIT only:** 720 **PP followed by MIT:** 360 **MIT followed by PP:** 360
Kanthack	**MI experience:** No pervious MIT experience **MI familiarization:** N.R. **MI check:** Three open-ended questions	**PETTLEP:** N.R. **MITG:** (a) Watching a 1 min video of great players from the NBA scoring free-throw baskets (b) MIT imagine the entire throw, from the movements of the body with a mechanical image of the arm and the trajectory of the ball through the air, emphasizing the ball being released and entering the hoop	**CG:** Participants were taken to another room for 4 min	**Session:** N.R. **Order:** MIT before PP	**Location:** Room off the basketball court, less than 20 m from the basket **Position:** Sitting	**Supervision:** Yes **Instructions:** N.R.	**Mode:** N.R. **Perspective:** N.R. **Eyes:** Closed	**Total:** 1 **Duration:** 3 min	**MITS:** N.R. **Total trials:** N.R.
Mohammadhasani	**MI experience:** N.R. **MI familiarization:** N.R. **MI check:** N.R.	**PETTLEP:** N.R. **MITG:** (a) PP of the kata skill with systematically increasing contextual interference for five sessions and five attempts each session in groups + MIT (b) MIT participants listened to the instructor and imagined that they were performing the movement patterns step by step	**PP1:** Participants physically practiced the kata skill with systematically increasing contextual interference for five sessions and five attempts each session in groups **PP2:** Participants practiced the kata skill for five sessions and five attempts each session in groups	**Session:** N.R. probably group **Order:** MIT before PP	**Location:** N.R. **Position:** Sitting on ground	**Supervision:** Yes **Instructions:** Live, acoustic Instructor loudly narrated the movement pattern of the kata skill step by step	**Mode:** N.R. **Perspective:** N.R. **Eyes:** Closed	**Total:** 5 **Duration:** N.R.	**MITS:** 5 **Total trials:** 25
Norouzi	**MI experience:** N.R. **MI familiarization:** N.R. **MI check:** N.R. and 2 open-ended questions	**PETTLEP:** Yes If participants wished to modify their imagery activity, such modifications were incorporated in subsequent imagery sessions (learning). Two different MIT groups **MIT1:** Internal PETTLEP + PP **MIT2:** External PETTLEP + PP	**PP:** Participants practiced physically only	**Session:** Group **Order:** MIT before PP	**Location:** Football field **Position:** N.R.	**Supervision:** Yes, once a week **Instructions:** Verbal, audio	**Mode:** N.R. **Perspective:** N.R. **Eyes:** N.R.	**Total:** 12 3 MITS/wk lasting 10 × 2 min = 20 min **Duration:** 4 weeks	**MITS:** N.R. **Total trials:** N.R.
Porretta	**MI experience:** N. R. **MI familiarization:** Explanation until participants understood MIT and MIT for 4 trials **MI check:** Participants were asked how they actually imaged the task after familiarization and after each practice day	**PETTLEP:** N.R. **MITG:** (a) MIT with seeing and feeling swinging the bat and hitting the string themselves (b) PP of swinging the bat and hitting the string	**PP:** Participants were swinging the bat and hitting the string + were solving mathematical problems in between	**Session:** Individual **Order:** MIT before PP	**Location:** N.R. **Position:** N.R.	**Supervision:** N.R. **Instructions:** Live, acoustic	**Mode:** Visual and kinesthetic **Perspective:** N.R. **Eyes:** Closed	**Total:** 5 **Duration:** 5 days	**MITS:** 25 × 4 mental trials **Total trials:** 100
Quinton	**MI experience:** N.R. **MI familiarization:** Participants were given a stimulus–response training in the first session to help them be more aware of what they were seeing and feeling during their imagination **MI check:** N.R.	**PETTLEP:** Yes, partially Participants were dressed in soccer kit, foot placed on the ball, usual environment (same gymnasium), changing session content **MITG:** (a) PP of soccer performance task: dribbling and passing (b) MIT of soccer performance task: dribbling and passing, MITS were designed as a layered-PETTLEP approach, with more elements introduced as the intervention progressed.	**CG:** PP + participants received sport-specific nutritional advice	**Session:** N.R. **Order:** PP before MIT	**Location:** Gymnasium **Position:** Standing, foot placed on ball, dressed in soccer kit	**Supervision:** N.R. **Instructions:** Live, acoustic	**Mode:** according to personal preferences **Perspective:** according to personal preferences **Eyes:** according to personal preferences	**Total:** 10 Two MITS/wk **Duration:** 5 weeks	**MITS:** N.R. **Total trials:** N.R.
Screws	**MI experience:** N.R. **MI familiarization:** Imagery training orientation: Investigator discussed meaning of MIT and explained how to use MIT for motor task enhancement Participants were given MIT activities to acquaint them with MIT procedures. **MI check:** N.R.	**PETTLEP:** N.R. **MITG:** (a) PP of 20 trials on peg board or pursuit rotor game (b) MIT of 20 mental trials on peg board or pursuit rotor game	Two control groups: **CG1:** Participants physically practiced 20 trials on peg board or pursuit rotor game + made different geometric shapes **CG2:** no PP or MIT at any time, spent same amount of time with researcher	**Session:** N. R. **Order:** N. R.	**Location:** N. R. **Position:** N. R.	**Supervision:** N.R. **Instructions:** N. R.	**Mode:** N.R. **Perspective:** N.R. **Eyes:** N.R.	**Total:** 8 Five days/wk until prescribed number of sessions were completed **Duration:** 8 MITS, total 164 min	**MITS:** 20 **Total trials:** 160
Seif-Barghi	**MI experience:** Little experience **MI familiarization:** Introduction session for defining and explanation of sport imagery, its application in soccer. Participants completed exercises to develop external and internal imagery, real time speeds of images and create images applying all senses. **MI check:** Feedback sessions at the end of each MITS Weekly interviews instantly before MIT Randomly asked questions about training course	**PETTLEP:** N.R. **MITG:** (a) MIT with specific and general cognitive elements: Participants were remembered to focus on foot movements, angles, velocity, point of the ball stroke, kick force and following the ball toward the recipient (b) Normal PP training and match activities	**CG:** N.R. = neutral task group’ + normal training and match activities	**Session:** N.R probably group **Order:** N.R.	**Location:** Quiet room in their football clubs **Position:** N.R.	**Supervision:** Yes **Instructions:** N.R.	**Mode:** N.R. **Perspective:** N.R. **Eyes:** Participants were recommended to start MIT with eyes closed. With increasing experience they could continue with eyes either open or closed	**Total:** 8 One MITS/wk lasting 10–15 min = 150 min MIT should be used on daily basis **Duration:** 8 weeks	**MITS:** N.R. **Total trials:** N.R.
Simonsmeier	**MI experience:** Assessed at BL **MI familiarization:** Athletes participated in a 20 min workshop, to facilitate understanding of MIT importance for motor learning Brief introduction to imagery script **MI check:** Four-item questionnaire post intervention, MIT diary for participants and trainers	**PETTLEP:** Yes **MITG:** (a) MIT—participants used kinesthetic cues and imaged the movement at different speed (one time slower compared to physical execution of the task and two times in real-time; timing) and always from an internal perspective (b) Normal PP training	**CG:** normal PP training	**Session:** N.R. probably individual **Order:** N.R.	**Location:** Regular training gym wearing regular clothes **Position:** N.R.	**Supervision:** N.R., probably unsupervised **Instructions:** Pre-recorded audio script	**Mode:** Visual and kinesthetic **Perspective:** Internal Eyes: N.R.	**Total:** 16 Four MITS/wk lasting 5 min = 80 min **Duration:** 4 weeks	**MITS:** Three mental trials **Total trials:** 48
Surburg	**MI experience:** N. R. **MI familiarization:** Preparing subjects MIT with multiple trials of closing the eyes and rehearse task **MI check:** Questions regarding MIT content post-intervention	**PETTLEP:** N.R. **MITG:** (a) MIT of the underhand baseball throw (b) PP of the underhand baseball throw	Two different PP groups were participants practiced an underhand throw with the non-preferred hand: **PP1:** Low cognitive condition: experimenter stood next to the right side of the target **PP2:** High cognitive condition: experimenter served as base runner and participant had to toss the ball to the next base	**Session:** N.R. **Order:** MIT before PP	**Location:** N.R. **Position:** N.R.	**Supervision:** N. R. **Instructions:** N.R.	**Mode:** N. R. Perspective: N.R. **Eyes:** Closed	**Total:** N.R. **Duration:** 1 week	**MITS:** N.R. **Total trials:** N.R.
Takazono	**MI experience:** N.R. **MI familiarization:** N.R. **MI check:** N.R.	**PETTLEP:** N.R. **MITG:** (a) MIT of 180 trials of the experimental task: holding a plastic block with the index and thumb fingers and inserting it into a support (b) PP of 60 physical trials of the task	Two different control groups **PP:** 240 physical trials of the experimental task **CG:** 180 mental trials of another visual rotation + 60 physical trials of the experimental task	**Session:** N.R., probably individual **Order:** MIT before PP	**Location:** N.R. **Position:** Seated in a chair in front of a table, starting with the palm of the right hand downwards resting on the starting point	**Supervision:** N. R., probably yes **Instructions:** Live, acoustic	**Mode:** N.R., ‘Imagine this movement, thinking of all the sensations it provides’ **Perspective:** N.R. **Eyes:** Closed	**Total:** 1 **Duration:** 1 day	**MITS:** 240 **Total trials:** 240
Wilson	**MI experience:** N.R. **MI familiarization:** N.R. **MI check:** N.R.	**PETTLEP:** N.R. **MITG:** Software-based MIT Dynamic stimulus materials were presented in increasing complexity: (a) visual imagery exercises involving predictive timing (b) relaxation protocol and mental preparation (c) visual modeling of fundamental motor skills = watching videos (d) MIT of skills from external perspective (e) MIT of skills from internal perspective (f) PP	Two different control groups **PP:** Traditional perceptual-motor training of the experimental task **NP:** wait-list control	**Session:** Individual **Order:** MIT before PP	**Location:** N.R. **Position:** N.R., probably sitting in front of a computer screen	**Supervision:** Yes **Instructions:** live	**Mode:** N.R., probably kinesthetic **Perspective:** External before internal **Eyes:** N.R.	**Total:** 5 1 MITS/wk **Duration:** 5 weeks	**MITS:** 50 **Total trials:** N.R., probably 250

**Legend:** CG = control group; FOS = finger opposition sequence; min = minute; MI = motor imagery; MIT = motor imagery training; MITG = motor imagery training group; MITS = motor imagery training session; NP = no practice; N.R. = not reported; PETTLEP = acronym for physical, environment, timing, task, learning, emotion, perspective; PP = physical practice; sec = seconds; TS = training session; WBD = weight bearing distribution; wk = week.

### 2.4. Data Analysis

#### Primary Outcomes

Data were analyzed using the Review Manager 5 software [95] and were pooled for meta-analysis when we considered studies to be sufficiently similar in terms of participants, interventions, comparisons, and outcomes. The weighted standardized mean differences (SMD) and their corresponding 95% confidence intervals were extracted from the individual studies and were visualized in forest plots. The analysis included the main outcome measure for motor function as specified by the original study investigator. For the meta-analysis, it was specified to analyze the results using the random-effects model with the inversed-variance method due to likely heterogeneity between studies. To test for heterogeneity, the Q-test with its corresponding degrees of freedom (df) and *p*-value for an alpha level of 5% was used. Higgins’ I^2^ statistic [96] was chosen as a measure of heterogeneity, indicating how much of the total observed variance can be explained by the true variation between studies and to measure the actual dispersion of variance [78]. Further analyses were planned if data were sufficient for a sensitivity analysis or an analysis of secondary outcomes.

## 3. Results

Our searches identified 7238 references. After removal of duplicates, we screened titles and abstracts, and identified 79 potentially eligible references for full-text reading. All available abstracts were in English. After reading the full texts of the obtained references, we selected 22 studies for inclusion in this review and meta-analysis. The procedure of the search is depicted in the PRISMA study flowchart (Figure 1).

Two reviewers (VZ and CSA) separately examined whether the relevant studies fitted the population, intervention, comparison, outcome and study design (PICOS) strategy of our research question. Two authors were contacted for missing data. Disagreement of selected full texts was resolved with mutual consent. The kappa statistic after full text screening was 0.81. The reviewers could not agree on four studies and therefore a third reviewer (FB) was consulted to decide on the studies’ eligibility resulting in two studies that were included and two that were excluded.

### 3.1. Characteristics of the Included Studies

All 22 studies included in this review, identified as randomized controlled trials, are listed with their characteristics in Table 2. Included studies were conducted in ten different countries with six studies coming from Brazil and were published between 1995 and 2021 covering a period of 26 years. The sample sizes ranged from 18 to 136 participants with a total of 934 and a mean age ranging from nine to 18 years. Overall, the studies with stated gender distribution included 40.7% female and 59.3% male children or adolescents. For six studies, a gender distribution was not reported. Two studies included mentally retarded adolescents [83,86], one study included children with motor coordination difficulties [94] and one study children with mild mental disabilities [89]. The majority of studies included healthy pupils with 10 studies focusing on children practicing sports, e.g., tennis, gymnastic, basketball, and dance. The motor tasks under investigation varied greatly. Eight out of 22 studies focused on the upper extremity with a throwing task (e.g., basketball, tennis, baseball). Soccer passing performance was evaluated in three studies [86,88,90] and four studies focused on whole body movements (e.g., kata skill, the cast to hand-stand on bars, vertical jumps, elevé movement (‘…a core dance movement during which the dancer rises up while bearing weight on the fore-feet’. Thomas (2003) from Abraham, Dunsky, Dickstein, [73], page 2)) [73,76,85,91].

Study descriptions revealed inhomogeneity with respect to the intervention setting which was not described in three reports. Almost all studies performed a pre- and posttest for the motor task under investigation that was evaluated by a blinded assessor, judge or coach in five studies only. Fifteen of the 22 included studies did not report on blinding. Furthermore, only two studies performed a follow up retention test after ten or 28 days [74,83]. A participant flow chart was provided in two out of 22 studies [78,90] and dropouts were reported in four studies [73,77,90,91].

### 3.2. Characteristics of Included Motor Imagery Training Interventions

Table 3 provides an overview about the trial-related characteristics of the studies included. All studies used MIT alone or associated with action observation (AO) or PP in the experimental groups. Three studies applied the PETTLEP approach [76,78,86]. The PETTLEP model of motor imagery provides guidance for the effective delivery of such interventions [93]. According to this model, seven key components are to be considered when developing an intervention (Physical, Environment, Task, Timing, Learning, Emotion, and Perspective).

Before starting an MIT, participant’s experience with MIT was evaluated in seven out of 22 studies. A MI familiarization or introduction session before MIT was reported in seven studies and a manipulation check during or after the MIT intervention to ensure participants’ engagement in MIT was described in 14 studies with a focus on open-ended questions regarding the MIT content. Further important MIT session elements can be described as follows:

- MIT as individual one-to-one session or group session was reported in four studies only (2 × group session, 2 × one-to-one session),
- A combination of MIT with PP was reported in 16 studies with an equal distribution whether MIT was performed before or after PP,
- Nine studies reported the location of the MIT and eight studies reported the position of the participant during MIT,
- Supervision during MIT session was reported and provided in eleven studies,
- Used instructions to guide the participants in their MIT was stated in ten studies using mainly live and acoustic instructions,
- The MI mode was described in seven studies reporting both visual and kinesthetic modes, which is similar to MI perspective. MI perspectives (internal, external) were described in nine studies. Both MIT session elements, mode and perspective, were used separately or in combination,
- Authors reported in ten studies whether participants had open or closed eyes during MIT,
- Surprisingly, participants were evaluated regarding their MI ability in twelve studies only. Authors used different standardized (MIQ, MIQ-R, MIQ-RS, MIQ-C) or customized MI ability questionnaires or EMG recordings,
- Temporal parameters regarding MIT sessions can be summarized as follows: Number of total MIT sessions varied between one and 24 with an intervention duration between one day and eight weeks. One MIT session took about three to 34 min, while between three and 80 MI trials were performed, summing up to 720 MI trials over one MIT intervention period.

Overall, the reporting of the MIT elements and temporal parameters was incomplete, which reduces the chance of a high replicability or successful transfer to the routine use of MIT in children and adolescents in sports and health care.

### 3.3. Risk of Bias

**Figure 2:**
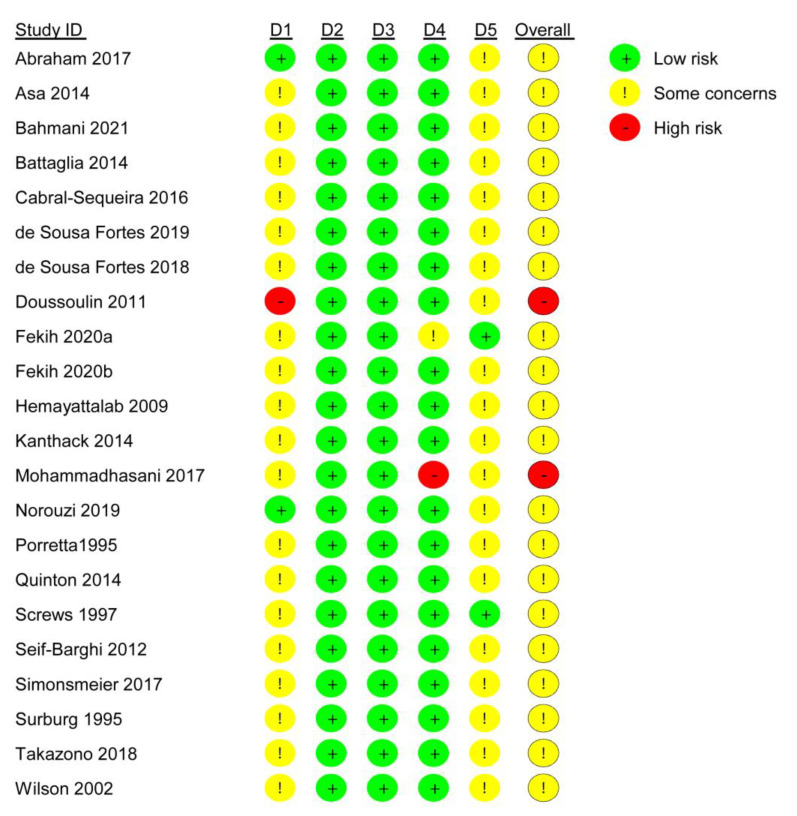
Risk of bias rating for each study. Legend: D1 = Randomization process, D2 = deviations from intended interventions, D3 = missing outcome data, D4 = measurement of the outcome, D5 = selection of the reported results.

**Figure 3:**
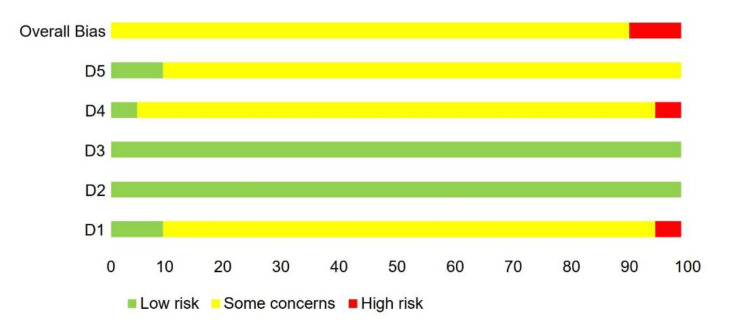
Risk of bias rating within domains Domain 1 to Domain 5 as percentage of all studies. Legend: D1 = Randomization process, D2 = deviations from intended interventions, D3 = missing outcome data, D4 = measurement of the outcome, D5 = selection of the reported results.

The results of the RoB evaluation (low, high, some concerns) regarding the six domains (randomization process, deviations from intended interventions, missing outcome data, measurement of the outcome, selection of the reported results, overall bias) are depicted in Figure 2 and Figure 3. The RoB evaluation was exclusively or predominantly categorized as low risk in the domains deviations from the intended interventions, missing outcome data and measurement of the outcome. Moreover, evaluation of the studies prevailingly revealed some concerns for Randomization process and Selection of the reported result. Only two studies were of high risk for overall bias [80,85]. A problematic baseline imbalance was found for the study of Doussoulin et al. (2011), whereas Mohammadhasani et al. (2017) revealed a likely influence on the assessments by knowledge of the intervention. None of the studies was judged low risk overall. We identified no information associated with other potential sources of bias.

### 3.4. Primary Outcomes-Effect of Motor Imagery Training Interventions

An overview of the findings of every included study in our review is provided in Table 2, indicating a general positive effect of MIT compared to a control group. However, the combination of MIT and PP was more successful than MIT only. Only three out of 22 studies reported no significant differences between MIT group (MITG) compared to a control group [73,84,88]. Due to the diversity of the studies’ methodologies regarding intervention protocols, participants and outcome parameters, only two out of 22 included studies could be considered for the meta-analysis regarding the effect of MIT on motor learning [78,82]. Both studies with 66 participants in total investigated the effect of MIT versus no intervention (watching videos) in young tennis players on the tennis service performance. Tennis service performance was the product calculated from measurements of the ball stroke velocity and accuracy. 

**Figure 4:**
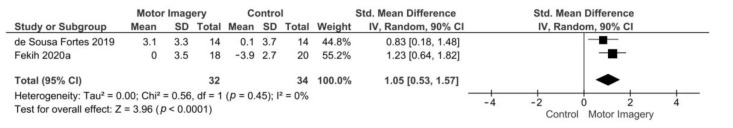
Effect of motor imagery training on tennis service accuracy versus watching videos as control intervention.

For the evaluation of the effect of MIT on the accuracy (Figure 4), the weighted SMD was 1.05 (95% CI; 0.53 to 1.57; Z = 3.96; *p* < 0.0001). Heterogeneity was very low (I^2^ = 0.00%; Q = 0.56; df = 1, *p* = 0.45). The variance of the distribution of the effect sizes in this sample of the two studies was T^2^ = 0.00.

**Figure 5:**
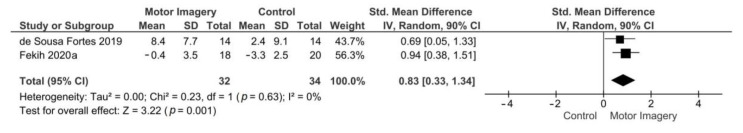
Effect of motor imagery on tennis stroke velocity versus watching videos as control intervention.

For the evaluation of the effect of MIT on tennis stroke velocity (Figure 5), the weighted SMD was 0.83 (95% CI; 0.33 to 134; Z = 3.22; *p* = 0.001). Heterogeneity was very low (I^2^ = 0.00%; Q = 0.23; df = 1, *p* = 0.63). The variance of the distribution of the effect sizes in this sample of the two studies was T^2^ = 0.00.

**Figure 6:**
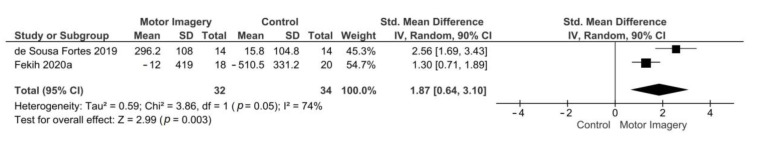
Effect of motor imagery training on tennis service performance versus watching videos as control intervention.

For the evaluation of the effect of MIT on the tennis service performance (the product of accuracy and speed velocity, Figure 6), the weighted SMD was 1.87 (95% CI; 0.64 to 3.10; Z = 2.99; *p* = 0.003). Heterogeneity was higher for this combined parameter (I^2^ = 0.74%; Q = 3.86; df = 1, *p* = 0.05). The variance of the distribution of the effect sizes in this sample of the two studies was T^2^ = 0.59.

### 3.5. GRADE Evidence Profile Table

After the evidence was summarized, small sample sizes, the width and overlap of confidence intervals, heterogeneity and generalizability were taken into consideration. Two reviewers (ZS and FB) created a GRADE evidence profile table (Table 4) to present key information [97]. Both examiners cross-checked each other’s assessments. Disagreements were solved by discussion.

### 3.6. Further Analyses

Subgroups for secondary analyzes could not be defined due to the lack of standardized evaluation at every measurement event (e.g., imagery ability was not always assessed before and at the end of an MIT). Imagery ability was mainly used as a screening criterion or to distinguish between high-level and low-level images. Furthermore, a sensitivity analysis (e.g., does not include a study with a high RoB or studies that included videos as MIT preparation) was not conducted because only two studies were included in the primary meta-analysis.

## 4. Discussion

This systematic review aimed to assess the effects of a MIT with or without PP on motor learning of various motor tasks to be trained, compared with different measures in the control condition. Physical practice or watching videos of a topic not related to MIT were the most applied interventions in the control groups. The number of included studies was 22, which involved 934 children and adolescents in total. None of the included studies reported adverse events. Only two studies could be included in a meta-analysis that revealed a high training effect with a low certainty of the evidence for the outcomes.

### 4.1. Motor Imagery Training Interventions

MIT use varied among intervention groups, with a majority of them using MIT in combination with PP. Some authors additionally used videos to prepare the mental simulation and illustrate the task to be imagined [76,84,94]. This might be perceived as a significant deviation from the other studies, as it may already correspond to a combined approach of AO and MI (AOMI). Similar to MI, AO also requires the activation of brain areas that are involved in generating body movements [98,99]. There are a large number of clinical trials that have investigated the use of either MI or AO alone in neurorehabilitation, but studies on the combination are still quite rare, especially in children or adolescents. Only recently; however, two studies reported on the positive effect of AOMI in children with DCD [100,101]. Scott et al. (2020) reported that effect also for typically developing children without DCD and found a significant enhancement in the outcome measure compared to the usage of MI alone [101].

Surprisingly, the PETTLEP approach was used in only three of the studies for an enhancement of the MI intervention [76,86,88]. In this respect, there was some inhomogeneity between the studies in terms of intervention design. Holmes and Collins (2001) developed the PETTLEP approach as a seven-point evidence-based checklist [102]. The authors’ aim was to enhance the efficacy of MIT interventions by using a systematic approach for their design, research and reporting. So far, the promising potential of the PE TTLEP model was widely neglected in the MIT interventions in the included studies.

Temporal parameters reporting (e.g., regarding MIT duration and MI trials per session) was sparse. However, if reported, the parameters were comparable with MIT reviews from other disciplines [70,103]. Focusing on children, some of the MIT sessions (3 min) and MIT interventions might be shorter (one to several days).

In our review, five studies included children starting at the age of nine and children with different levels of sports proficiency. Caeyenberghs et al. (2009) investigated the development of movement imagery over the childhood between the age of seven and twelve [104]. Authors found a relationship between MI and a motor skill becoming stronger with age. Furthermore, Mulder et al. (2007) also highlighted a possible relationship between level of physical activities and MI capacity and MI perspective selection [105]. Therefore, the design of MIT parameters (e.g., internal or external MI perspective), visual or kinesthetic mode, and the MI familiarization might differ for different age groups and should be considered for further research.

Unexpectedly, participants’ MI ability was not evaluated systematically in all 22 studies. MI is a multidimensional construct and the ability to create and manipulate a mental image is an essential criterion for an efficient MIT intervention. It is; thus, advised to use several assessments to evaluate the quality of participants’ MI ability [106,107]. Furthermore, information whether participants are novices to the MIT technique or already professional users should be evaluated and reported.

### 4.2. Methodology of the Included Studies

The quality of description of the study design and intervention are essential aspects in the evaluation process of RCTs. The CONSORT (2010) guideline was introduced to improve the reporting of RCTs [108]. For the included studies in this review, we found that the CONSORT recommendations were not fully implemented by any report, in part of course because the studies were conducted before the guideline was published. A flow diagram for instance that displays the progress of all participants through the trial as recommended was only provided in two reports [78,90]. The partially inadequate description of the studies according to CONSORT influenced the risk of bias evaluation due to missing information. For domain 1, which concerns the randomization process, the lack of information on the question whether the allocation sequence was concealed until participants were enrolled and assigned to intervention (RoB 1.2) could automatically only result in some concerns or an even lower judgment. Insufficient information about the awareness of the assessors regarding the intervention received for the RoB rating process in domain 4 also led to some concerns. A detailed study protocol would have been beneficial.

The TIDieR checklist was developed to improve the quality of the intervention description and thus its replicability [109]. It is an extension of the CONSORT 2010 statement with the aim of providing a practical tool for authors, reviewers and readers. None of the studies followed or complied with the TIDieR guideline for reporting an intervention. Additionally, in terms of methodological quality and interventional approach, it is worth noting that no study included a follow-up measurement to evaluate a potential long-term effect.

### 4.3. Strengths and Limitations

The underlying systematic review process of this report may have been influenced by the approach of searching English-language databases only. However, the language-based confounding factor is probably mitigated by the fact the main literature sources are English-speaking peer-reviewed journals. The composition of the search strategy was based on recently published Cochrane reviews and other review protocols in the field of MI and mental practice [31,66], and the database searches itself were conducted by a professional research librarian providing a comprehensive search and detailed knowledge of different databases with a medical focus. The systematic searches were conducted in seven databases and one trial registry covering different disciplines, for example, sports, psychology and populations (e.g., healthy children, children with a high proficiency in a sport or with a coordination disorder). The inclusion of only published, peer-reviewed data was due to the attempt to make a truly reliable statement about the effectiveness of MIT.

A further strength is that we were able to include studies from different parts of the world and thus reports on trials conducted in different cultural settings. Included studies represent children from different countries and continents (North and South America, the Middle East, Europe and Australia) with an emphasis on South America.

We performed important and recommended study appraisals and classifications (e.g., RoB, GRADE), to provide the reader a comprehensive evidence evaluation. Additionally, we could pool results from two studies from Sports focusing on tennis stroke performance in a meta-analysis revealing a high MIT effect. Here; however, the results of I^2^ should be interpreted with caution. Due to the small number of pooled studies, I^2^ might be biased and under- or overestimate the true level of heterogeneity [110].

Finally, for the interested clinician and researcher, we extracted and described conducted MIT interventions into detail and included important MIT elements and temporal parameters.

### 4.4. Implications for Further Research

The promising results of our systematic review encourage further and intensive research in the field of MIT in children and adolescents. (1) We recommend comprehensive investigations regarding MIT session elements and their temporal parameters in relation to age. (2) Further research should include a routinely evaluation of participants’ MI ability quality with standardized assessment tools. (3) A MI introduction session at the beginning of the MIT intervention is advised to ensure similar level of MI knowledge of all participants. (4) To evaluate participants’ engagement in MIT, a MI check on a regular basis should be integrated. Based on our results, open-ended questions might be helpful. (5) Longer follow-up periods to evaluate MIT’ retention would be desired. (6) Finally, a detailed reporting based on the CONSORT, PETTLEP and TIDieR checklists is highly recommended to ensure replicability and transfer to clinical use of MIT in children and adolescents.

## 5. Conclusions

With regard to children and adolescents, the method of motor imagery training has not received much attention in research in the recent past. Only a few high quality RCTs exist and reporting on motor imagery training elements and temporal parameters should be improved. However, there are indications that motor imagery training might have a high potential for healthy and impaired children and adolescents if combined with physical practice to enhance motor learning in sports and in general. With regard to the treatment of children with neurological disorders using MI, a further three ongoing studies registered on clinicaltrials.org (accessed on 15 April 2021) have been identified, and will hopefully provide new results in this field. There is also a growing body of literature concerning the effect of MI in children and adolescents in the treatment of psychological disorders such as stress or anxiety, which; however, was not within the scope of this review.

## Figures and Tables

**Table 1:** Search terms and strategy of the Embase session.

**No.**	**Query**	**Results**
#5	#1 AND #2 AND #3 NOT [conference abstract]/lim NOT ([animals]/lim NOT [humans]/lim)	1388
#4	#1 AND #2 AND #3	1695
#3	‘controlled clinical trial’/exp OR crossover*:ti,ab,kw OR ‘cross-over*’:ti,ab,kw OR placebo*:ti,ab,kw OR sham:ti,ab,kw OR (((single OR double) NEAR/2 blind*):ti,ab,kw) OR random*:ti,ab,kw OR allocat*:ti,ab,kw OR factorial*:ti,ab,kw OR assign*:ti,ab,kw OR (((clinical OR controlled) NEAR/2 (trial* OR stud*)):ti,ab,kw) OR trial:ti	3,145,824
#2	‘motor imagery’/exp OR ‘motor imagery training’/exp OR ‘imagery’/exp OR ‘imagination’/exp OR (((motor OR locomot* OR mental OR kinesthetic* OR kinaesthetic* OR movement*) NEAR/2 (imag*OR simulation* OR ideation* OR visual*)):ti,ab,kw) OR (((mental* OR cognitive* OR covert*) NEAR/2 (movement* OR rehears* OR imag* OR practic* OR practis* OR training* OR represent*OR stimulation* OR ideation* OR visual*)):ti,ab,kw) OR imagery:ti,ab,kw OR imagining:ti,ab,kw OR imagination*:ti,ab,kw	64,715
#1	‘child’/exp OR ‘adolescent’/exp OR ‘pediatrics’/exp OR ‘cerebral palsy’/exp OR ‘developmental coordination disorder’/exp OR child*:ti,ab,kw OR boy:ti,ab,kw OR boys:ti,ab,kw OR girl*:ti,ab,kw OR pediatric*:ti,ab,kw OR paediatric*:ti,ab,kw OR adoles*:ti,ab,kw OR teen*:ti,ab,kw OR ‘preschool*’:ti,ab,kw OR (((cerebral OR brain OR spastic) NEAR/2 (palsy OR paralys* OR pares* OR diplegia)):ti,ab,kw) OR ‘developmental coordination disorder*’:ti,ab,kw OR dcd:ti,ab,kw	4,523,492

**Legend:** ab = abstract; dcd = developmental coordination disorder; exp = exploded; kw = keyword heading; lim = limit; ti = title.

**Table 4:** Evidence profile table.

Certainty Assessment							No. of Patients		Effect		Quality of Evidence	Importance
No. of Studies	Study Design	Risk of Bias	Inconsistency	Indirectness	Imprecision	Other Considerations	MI	Control	Relative (95% CI)	Absolute (95% CI)		
**Accuracy (Follow-up range 3 to 4 weeks)**												
2	RCT	serious ^a^	not serious	not serious ^b^	serious ^c^	none	32	34	-	SMD 1.05 (0.53, 1.57)	⨁⨁◯◯ LOW	not important
**Ball stroke speed (Follow-up range 3 to 4 weeks)**												
2	RCT	serious ^a^	not serious	not serious ^b^	serious ^c^	none	32	34	-	SMD 0.83 (0.33, 1.34)	⨁⨁◯◯ LOW	not important
**Tennis service performance (Follow-up range 3 to 4 weeks)**												
2	RCT	serious ^a^	serious ^d^	not serious ^b^	serious ^c^	none	32	34	-	SMD 1.87 (0.64, 3.10)	⨁◯◯◯ VERY LOW	not important

RCT = randomized controlled trial, SMD = standard mean difference, CI = confidence interval. ^a^ = some concerns regarding randomization process and selection of the reported results, ^b^ = indirectness of population, only male tennis players investigated, ^c^ = serious imprecision (i.e., total number of participants <300 for each outcome), ^d^ = significant statistical heterogeneity.

## Data Availability

Data used for result tables, forest plots, RoB and GRADE evaluation can be requested from the first author upon request.

## References

[1] Schott N., Haibach-Beach P., Knöpfle I., Neuberger V. (2021). The effects of visual impairment on motor imagery in children and adolescents. Res. Dev. Disabil..

[2] Guillot A., Di Rienzo F., MacIntyre T., Moran A.P., Collet C. (2012). Imagining is Not Doing but Involves Specific Motor Commands: A Review of Experimental Data Related to Motor Inhibition. Front. Hum. Neurosci..

[3] Munzert J., Lorey B., Zentgraf K. (2009). Cognitive motor processes: The role of motor imagery in the study of motor representations. Brain Res. Rev..

[4] Conson M., Mazzarella E., Trojano L. (2013). Developmental changes of the biomechanical effect in motor imagery. Exp. Brain Res..

[5] Jackson P., Lafleur M.F., Malouin F., Richards C.L., Doyon J. (2001). Potential role of mental practice using motor imagery in neurologic rehabilitation. Arch. Phys. Med. Rehabil..

[6] Jeannerod M. (1994). Motor representations and reality. Behav. Brain Sci..

[7] Sharma N., Pomeroy V., Baron J.-C. (2006). Motor Imagery: A backdoor to the motor system after stroke?. Stroke.

[8] Kilteni K., Andersson B.J., Houborg C., Ehrsson H.H. (2018). Motor imagery involves predicting the sensory consequences of the imagined movement. Nat. Commun..

[9] Ridderinkhof K.R., Brass M. (2015). How Kinesthetic Motor Imagery works: A predictive-processing theory of visualization in sports and motor expertise. J. Physiol..

[10] Decety J., Perani D., Jeannerod M., Bettinardi V., Tadary B., Woods R., Mazziotta J.C., Fazio F. (1994). Mapping motor representations with positron emission tomography. Nat. Cell Biol..

[11] Decety J. (1996). The neurophysiological basis of motor imagery. Behav. Brain Res..

[12] Roth M., Decety J., Raybaudi M., Massarelli R., Delon-Martin C., Segebarth C., Morand S., Gemignani A., Décorps M., Jeannerod M. (1996). Possible involvement of primary motor cortex in mentally simulated movement. NeuroReport.

[13] Fleming M., Stinear C., Byblow W. (2009). Bilateral parietal cortex function during motor imagery. Exp. Brain Res..

[14] Grosprêtre S., Lebon F., Papaxanthis C., Martin A. (2016). New evidence of corticospinal network modulation induced by motor imagery. J. Neurophysiol..

[15] Cumming R.R.J. (2009). Imagery Intervention in Sport. Advances in Applied Sport Psychology: A Review.

[16] Simonsmeier B.A., Androniea M., Buecker S., Frank C. (2020). The effects of imagery interventions in sports: A meta-analysis. Int. Rev. Sport Exerc. Psychol..

[17] Driediger M., Hall C., Callow N. (2006). Imagery use by injured athletes: A qualitative analysis. J. Sports Sci..

[18] Schuster C., Glässel A., Scheidhauer A., Ettlin T., Butler J. (2012). Motor Imagery Experiences and Use: Asking Patients after Stroke Where, When, What, Why, and How They Use Imagery: A Qualitative Investigation. Stroke Res. Treat..

[19] Braun S., Kleynen M., Van Heel T., Kruithof N., Wade D., Beurskens A. (2013). The effects of mental practice in neurological rehabilitation; a systematic review and meta-analysis. Front. Hum. Neurosci..

[20] Zimmermann-Schlatter A., Schuster C., Puhan M.A., Siekierka E., Steurer J. (2008). Efficacy of motor imagery in post-stroke rehabilitation: A systematic review. J. Neuroeng. Rehabil..

[21] Cramer S.C., Orr E.L.R., Cohen M.J., LaCourse M.G. (2006). Effects of motor imagery training after chronic, complete spinal cord injury. Exp. Brain Res..

[22] Lebon F., Guillot A., Collet C. (2011). Increased Muscle Activation Following Motor Imagery During the Rehabilitation of the Anterior Cruciate Ligament. Appl. Psychophysiol. Biofeedback.

[23] Marusic U., Grosprêtre S., Paravlic A., Kovač S., Pišot R., Taube W. (2018). Motor Imagery during Action Observation of Locomotor Tasks Improves Rehabilitation Outcome in Older Adults after Total Hip Arthroplasty. Neural Plast..

[24] Cupal D.D., Brewer B.W. (2001). Effects of relaxation and guided imagery on knee strength, reinjury anxiety, and pain following anterior cruciate ligament reconstruction. Rehabil. Psychol..

[25] Christakou A., Zervas Y., Lavallee D. (2007). The adjunctive role of imagery on the functional rehabilitation of a grade II ankle sprain. Hum. Mov. Sci..

[26] Sordoni C., Hall C., Forwell L. (2000). The Use of Imagery by Athletes during Injury Rehabilitation. J. Sport Rehabil..

[27] Bucher L. (1993). The Effects of Imagery Abilities and Mental Rehearsal on Learning a Nursing Skill. J. Nurs. Educ..

[28] Doheny M.O. (1989). Effects of Mental Practice on Psychomotor Skills with Baccalaureate Nursing Students. Doctoral Dissertation.

[29] Immenroth M., Bürger T., Brenner J., Nagelschmidt M., Eberspächer H., Troidl H. (2007). Mental Training in Surgical Education. Ann. Surg..

[30] Fairweather M.M., Sidaway B. (1993). Ideokinetic Imagery as a Postural Development Technique. Res. Q. Exerc. Sport.

[31] Silva S., Borges L.R., Santiago L., Lucena L., Lindquist A.R., Ribeiro T. (2020). Motor imagery for gait rehabilitation after stroke. Cochrane Database Syst. Rev..

[32] Di Rienzo F., Debarnot U., Daligault S., Saruco E., Delpuech C., Doyon J., Collet C., Guillot A. (2016). Online and Offline Performance Gains Following Motor Imagery Practice: A Comprehensive Review of Behavioral and Neuroimaging Studies. Front. Hum. Neurosci..

[33] Féry Y.-A. (2003). Differentiating visual and kinesthetic imagery in mental practice. Can. J. Exp. Psychol. Rev. Can. Psychol. Exp..

[34] Guillot A., Collet C., Dittmar A. (2004). Relationship Between Visual and Kinesthetic Imagery, Field Dependence-Independence, and Complex Motor Skills. J. Psychophysiol..

[35] Guillot A., Collet C., Nguyen V.A., Malouin F., Richards C.L., Doyon J. (2009). Brain activity during visual versus kinesthetic imagery: An fMRI study. Hum. Brain Mapp..

[36] Callow N., Roberts R., Hardy L., Jiang D., Edwards M.G. (2013). Performance improvements from imagery: Evidence that internal visual imagery is superior to external visual imagery for slalom performance. Front. Hum. Neurosci..

[37] Wakefield C., Smith D. (2012). Perfecting Practice: Applying the PETTLEP Model of Motor Imagery. J. Sport Psychol. Action.

[38] Cumming J., Williams S. (2012). The Role of Imagery in Performance. The Role of Imagery in Performance.

[39] Goss S., Hall C., Buckolz E., Fishburne G. (1986). Imagery ability and the acquisition and retention of movements. Mem. Cogn..

[40] Mizuguchi N., Nakata H., Uchida Y., Kanosue K. (2012). Motor imagery and sport performance. J. Phys. Fit. Sports Med..

[41] Williams J., Thomas P.R., Maruff P., Wilson P. (2008). The link between motor impairment level and motor imagery ability in children with developmental coordination disorder. Hum. Mov. Sci..

[42] Spruijt S., van der Kamp J., Steenbergen B. (2015). Current insights in the development of childrenâ€™s motor imagery ability. Front. Psychol..

[43] Butson M.L., Hyde C., Steenbergen B., Williams J. (2014). Assessing motor imagery using the hand rotation task: Does performance change across childhood?. Hum. Mov. Sci..

[44] Spruijt S., van der Kamp J., Steenbergen B. (2015). The ability of 6- to 8-year-old children to use motor imagery in a goal-directed pointing task. J. Exp. Child Psychol..

[45] Molina M., Tijus C., Jouen F. (2008). The emergence of motor imagery in children. J. Exp. Child Psychol..

[46] Estes D. (2008). Young Children’s Awareness of Their Mental Activity: The Case of Mental Rotation. Child Dev..

[47] Cheng Y.-L., Mix K.S. (2014). Spatial Training Improves Children’s Mathematics Ability. J. Cogn. Dev..

[48] Hawes Z., Moss J., Caswell B., Poliszczuk D. (2015). Effects of mental rotation training on children’s spatial and mathematics performance: A randomized controlled study. Trends Neurosci. Educ..

[49] Funk M., Brugger P., Wilkening F. (2005). Motor processes in children’s imagery: The case of mental rotation of hands. Dev. Sci..

[50] Souto D.O., Cruz T.K.F., Fontes P.L.B., Batista R.C., Haase V.G. (2020). Motor Imagery Development in Children: Changes in Speed and Accuracy with Increasing Age. Front. Pediatr..

[51] Bhoyroo R., Hands B., Wilmut K., Hyde C., Wigley A. (2019). Motor planning with and without motor imagery in children with Developmental Coordination Disorder. Acta Psychol..

[52] Adams I.L.J., Steenbergen B., Lust J.M. (2018). Development of motor imagery ability in children with developmental coordination disorder—A goal-directed pointing task. Br. J. Psychol..

[53] Adams I.L.J., Smits-Engelsman B., Lust J.M., Wilson P., Steenbergen B. (2017). Feasibility of Motor Imagery Training for Children with Developmental Coordination Disorder—A Pilot Study. Front. Psychol..

[54] Errante A., Bozzetti F., Sghedoni S., Bressi B., Costi S., Crisi G., Ferrari A., Fogassi L. (2019). Explicit Motor Imagery for Grasping Actions in Children with Spastic Unilateral Cerebral Palsy. Front. Neurol..

[55] American Psychatric Association (2013). Diagnostic and Statistical Manual of Mental Disorders (DSM-5®).

[56] Deconinck F.J.A., De Clercq D., Savelsbergh G.J.P., Van Coster R., Oostra A., Dewitte G., Lenoir M. (2006). Visual contribution to walking in children with Developmental Coordination Disorder. Child Care Health Dev..

[57] Wilson P.H., Ruddock S., Smits-Engelsman B., Polatajko H., Blank R. (2012). Understanding performance deficits in developmental coordination disorder: A meta-analysis of recent research. Dev. Med. Child Neurol..

[58] Adams I.L., Lust J.M., Wilson P., Steenbergen B. (2014). Compromised motor control in children with DCD: A deficit in the internal model?—A systematic review. Neurosci. Biobehav. Rev..

[59] Gabbard C., Bobbio T. (2010). The Inability to Mentally Represent Action May Be Associated with Performance Deficits in Children With Developmental Coordination Disorder. Int. J. Neurosci..

[60] Williams J., Thomas P.R., Maruff P., Butson M., Wilson P.H. (2006). Motor, visual and egocentric transformations in children with Developmental Coordination Disorder. Child Care Health Dev..

[61] Hyde C., Fuelscher I., Buckthought K., Enticott P., Gitay M.A., Williams J. (2014). Motor imagery is less efficient in adults with probable developmental coordination disorder: Evidence from the hand rotation task. Res. Dev. Disabil..

[62] Deconinck F.J.A., Spitaels L., Fias W., Lenoir M. (2009). Is developmental coordination disorder a motor imagery deficit?. J. Clin. Exp. Neuropsychol..

[63] Reynolds J.E., Licari M.K., Elliott C., Lay B.S., Williams J. (2015). Motor imagery ability and internal representation of movement in children with probable developmental coordination disorder. Hum. Mov. Sci..

[64] Crajé C., van Elk M., Beeren M., van Schie H.T., Bekkering H., Steenbergen B. (2010). Compromised motor planning and Motor Imagery in right Hemiparetic Cerebral Palsy. Res. Dev. Disabil..

[65] Mutsaarts M., Steenbergen B., Bekkering H. (2006). Anticipatory planning deficits and task context effects in hemiparetic cerebral palsy. Exp. Brain Res..

[66] Barclay R.E., Stevenson T.J., Poluha W., Thalman L. (2011). Mental practice for treating upper extremity deficits in individuals with hemiparesis after stroke. Cochrane Database Syst. Rev..

[67] Suica Z., Platteau-Waldmeier P., Koppel S., Schmidt-Trucksaess A., Ettlin T., Schuster-Amft C. (2018). Motor imagery ability assessments in four disciplines: Protocol for a systematic review. BMJ Open.

[68] Lefebvre C., Glanville J., Briscoe S., Littlewood A., Marshall C., Metzendorf M., Noel-Storr A., Rader T., Shokraneh F., Thomas J., Higgins J.P.T., Thomas J., Chandler J., Cumpston M., Li T., Page M.J., Welch V.A. (2021). Chapter 4: Searching for and selecting studies. Cochrane Handbook for Systematic Reviews of Interventions Version 6.2 (Updated February 2021).

[69] Landis J.R., Koch G.G. (1977). The Measurement of Observer Agreement for Categorical Data. Biometrics.

[70] Schuster C., Hilfiker R., Amft O., Scheidhauer A., Andrews B., Butler J., Kischka U., Ettlin T. (2011). Best practice for motor imagery: A systematic literature review on motor imagery training elements in five different disciplines. BMC Med..

[71] Higgins J.P.T., Altman D.G., Gøtzsche P.C., Jüni P., Moher D., Oxman A.D., Savović J., Schulz K.F., Weeks L., Sterne J.A.C. (2011). The Cochrane Collaboration’s tool for assessing risk of bias in randomised trials. BMJ.

[72] Ryan R., Hill S. (2018). How to GRADE. Cochrane Consum. Commun. La Trobe Univ..

[73] Abraham A., Dunsky A., Dickstein R. (2017). The Effect of Motor Imagery Practice on Elevé Performance in Adolescent Female Dance Students: A Randomized Controlled Trial. J. Imag. Res. Sport Phys. Act..

[74] Asa S.K.D.P., Melo M.C.S., Piemonte M.E.P. (2014). Effects of Mental and Physical Practice on a Finger Opposition Task among Children. Res. Q. Exerc. Sport.

[75] Bahmani M., Babak M., Land W.M., Howard J.T., Diekfuss J.A., Abdollahipour R. (2021). Children’s motor imagery modality dominance modulates the role of attentional focus in motor skill learning. Hum. Mov. Sci..

[76] Battaglia C., D’Artibale E., Fiorilli G., Piazza M., Tsopani D., Giombini A., Calcagno G., di Cagno A. (2014). Use of video observation and motor imagery on jumping performance in national rhythmic gymnastics athletes. Hum. Mov. Sci..

[77] Cabral-Sequeira A.S., Coelho D.B., Teixeira L.A. (2016). Motor imagery training promotes motor learning in adolescents with cerebral palsy: Comparison between left and right hemiparesis. Exp. Brain Res..

[78] Fortes L.D., Almeida S.S., Nascimento Junior J.R., Vieira L.F., Lima-Júnior D., Ferreira M.E. (2019). Effect of motor imagery training on tennis service performance in young tennis athletes. Rev. Psicol. Deporte.

[79] Fortes L., Freitas-Júnior C.G., Paes P.P., Vieira L.F., Junior J.R.N., de Lima-Junior D., Ferreira M.E.C. (2020). Effect of an eight-week imagery training programme on passing decision-making of young volleyball players. Int. J. Sport Exerc. Psychol..

[80] Doussoulin A., Rehbein L. (2011). Motor imagery as a tool for motor skill training in children. Motricidade.

[81] Fekih S., Zguira M.S., Koubaa A., Ghariani I., Zguira H., Bragazzi N.L., Jarraya M. (2020). The Impact of a Motor Imagery-Based Training Program on Agility, Speed, and Reaction Time in a Sample of Young Tennis Athletes during Ramadan Fasting: Insights and Implications from a Randomized, Controlled Experimental Trial. Nutrients.

[82] Fekih S., Zguira M.S., Koubaa A., Masmoudi L., Bragazzi N.L., Jarraya M. (2020). Effects of Motor Mental Imagery Training on Tennis Service Performance during the Ramadan Fasting: A Randomized, Controlled Trial. Nutrients.

[83] Hemayattalab R., Movahedi A. (2010). Effects of different variations of mental and physical practice on sport skill learning in adolescents with mental retardation. Res. Dev. Disabil..

[84] Kanthack T.F.D., Bigliassi M., Vieira L.F., Altimari L.R. (2013). Efeito agudo da imagética no desempenho de lances livres e percepção de autoeficácia em atletas. Braz. J. Kinanthropometry Hum. Perform..

[85] Mohammadhasani F., Rostami R., Cheric M.C. (2017). The Effect of a Combined Practice Course of Mental and Physical Practice with Systematic Increase in Contextual Interference on Learning a Kata Skill. Ann. Appl. Sport Sci..

[86] Norouzi E., Hossini R.N.S., Afroozeh M.S., Vaezmosavi M., Gerber M., Puehse U., Brand S. (2019). Examining the Effectiveness of a PETTLEP Imagery Intervention on the Football Skill Performance of Novice Athletes. J. Imag. Res. Sport Phys. Act..

[87] Porretta D.L., Surburg P.R. (1995). Imagery and Physical Practice in the Acquisition of Gross Motor Timing of Coincidence by Adolescents with Mild Mental Retardation. Percept. Mot. Ski..

[88] Quinton M.L., Cumming J., Gray R., Geeson J.R., Cooper A., Crowley H., Williams S.E. (2014). A PETTLEP Imagery Intervention with Young Athletes. J. Imag. Res. Sport Phys. Act..

[89] Screws D.P., Surburg P.R. (1997). Motor Performance of Children with Mild Mental Disabilities after Using Mental Imagery. Adapt. Phys. Act. Q..

[90] Seif-Barghi T., Kordi R., Memari A.-H., Mansournia M.A., Jalali-Ghomi M. (2012). The Effect of an Ecological Imagery Program on Soccer Performance of Elite Players. Asian J. Sports Med..

[91] Simonsmeier B.A., Frank C., Gubelmann H., Schneider M. (2018). The effects of motor imagery training on performance and mental representation of 7- to 15-year-old gymnasts of different levels of expertise. Sport Exerc. Perform. Psychol..

[92] Surburg P.R., Porretta D.L., Sutlive V. (1995). Use of Imagery Practice for Improving a Motor Skill. Adapt. Phys. Act. Q..

[93] Takazono P.S., Teixeira L.A. (2018). Efeito da associação de prática imagética e física na aprendizagem motora em crianças. Braz. J. Kinanthropometry Hum. Perform..

[94] Wilson P.H., Thomas P.R., Maruff P. (2002). Motor Imagery Training Ameliorates Motor Clumsiness in Children. J. Child Neurol..

[95] (2020). Review Manager Web (RevMan 5).

[96] Higgins J.P.T., Thompson S.G. (2002). Quantifying heterogeneity in a meta-analysis. Stat. Med..

[97] (2020). GRADEpro GDT: GRADEpro Guideline Development Tool.

[98] Grezes J., Decety J. (2001). Functional anatomy of execution, mental simulation, observation, and verb generation of actions: A meta-analysis. Hum. Brain Mapp..

[99] Gerardin E., Sirigu A., Lehéricy S., Poline J.-B., Gaymard B., Marsault C., Agid Y., Le Bihan D. (2000). Partially Overlapping Neural Networks for Real and Imagined Hand Movements. Cereb. Cortex.

[100] Marshall B., Wright D., Holmes P., Williams J., Wood G. (2020). Combined action observation and motor imagery facilitates visuomotor adaptation in children with developmental coordination disorder. Res. Dev. Disabil..

[101] Scott M.W., Emerson J.R., Dixon J., Tayler M.A., Eaves D.L. (2020). Motor imagery during action observation enhances imitation of everyday rhythmical actions in children with and without developmental coordination disorder. Hum. Mov. Sci..

[102] Holmes P.S., Collins D.J. (2001). The PETTLEP Approach to Motor Imagery: A Functional Equivalence Model for Sport Psychologists. J. Appl. Sport Psychol..

[103] Guerra Z.F., Lucchetti A.L.G., Lucchetti G. (2017). Motor Imagery Training After Stroke: A Systematic Review and Meta-analysis of Randomized Controlled Trials. J. Neurol. Phys. Ther..

[104] Caeyenberghs K., Tsoupas J., Wilson P., Smits-Engelsman B.C.M. (2009). Motor Imagery Development in Primary School Children. Dev. Neuropsychol..

[105] Mulder T., Hochstenbach J., van Heuvelen M., Otter A.D. (2007). Motor imagery: The relation between age and imagery capacity. Hum. Mov. Sci..

[106] Collet C., Guillot A., Lebon F., MacIntyre T., Moran A. (2011). Measuring Motor Imagery Using Psychometric, Behavioral, and Psychophysiological Tools. Exerc. Sport Sci. Rev..

[107] Lequerica A., Rapport L., Axelrod B.N., Telmet K., Whitman R.D. (2002). Subjective and Objective Assessment Methods of Mental Imagery Control: Construct Validations of Self-Report Measures. J. Clin. Exp. Neuropsychol..

[108] Moher D., Hopewell S., Schulz K.F., Montori V., Gøtzsche P.C., Devereaux P.J., Elbourne D., Egger M., Altman D.G. (2010). CONSORT 2010 explanation and elaboration: Updated guidelines for reporting parallel group randomised trials. BMJ.

[109] Hoffmann T.C., Glasziou P.P., Boutron I., Milne R., Perera R., Moher D., Altman D.G., Barbour V., Macdonald H., Johnston M. (2014). Better reporting of interventions: Template for intervention description and replication (TIDieR) checklist and guide. BMJ.

[110] Von Hippel P.T. (2015). The heterogeneity statistic I2 can be biased in small meta-analyses. BMC Med Res. Methodol..

